# First and Second Level Haemoglobinopathies Diagnosis: Best Practices of the Italian Society of Thalassemia and Haemoglobinopathies (SITE)

**DOI:** 10.3390/jcm11185426

**Published:** 2022-09-15

**Authors:** Giorgia Mandrile, Susanna Barella, Antonino Giambona, Antonia Gigante, Michela Grosso, Silverio Perrotta, Saverio Scianguetta, Gian Luca Forni

**Affiliations:** 1 SSD Microcitemie—AOU S. Luigi Gonzaga—Orbassano, 10043 Turin, Italy; 2 SSD Talassemia-Ospedale Pediatrico Microcitemico A. Cao, 09121 Cagliari, Italy; 3 UOC di Ematologia e Malattie Rare del Sangue e degli Organi Emopoietici, A.O.O.R. Villa Sofia-Cervello, 90121 Palermo, Italy; 4 Società Italiana Talassemie d Emoglobinopatie (SITE), Fondazione per la Ricerca sulle Anemie ed Emoglobinopatie in Italia, For Anemia, 16125 Genoa, Italy; 5 Dipartimento di Medicina Molecolare e Biotecnologie Mediche, Università di Napoli Federico II, CEINGE-Biotecnologie Avanzate, AOU Federico II, 80138 Naples, Italy; 6 Ematologia ed Oncologia Pediatrica, D.A.I. Materno Infantile, AOU Università degli Studi della Campania “Luigi Vanvitelli”, 80138 Naples, Italy; 7 Centro della Microcitemia, Anemie Congenite e Dismetabolismo del Ferro—E.O. Galliera, 16128 Genoa, Italy

**Keywords:** diagnostics of hemoglobinopathies, prenatal diagnosis, Thalassemia, sickle cell disease, hemoglobinopathies

## Abstract

The purpose of this best practice paper is to review the current recommendations for the identification and prenatal diagnosis of hemoglobinopathies. Methods: The management committee of SITE selected and gathered a multidisciplinary team in order to formulate recommendations based on the available scientific evidence integrated with the opinions of experts, with the purpose of supporting clinicians. Results: We provide recommendations for first level tests (complete blood count, hemoglobin separation and iron balance), second level tests (molecular diagnosis) and prenatal diagnosis. Five Italian experts in hemoglobinopathies were consulted regarding the orientation of prenatal diagnosis, and for each indication, the degree of agreement among the experts has been specified. Conclusions: Best practice recommendations are the final outcome of this translational research and allow transfer to daily clinical practice.

## 1. Introduction

Hemoglobinopathies are the most frequent genetic diseases worldwide, and Italy displays a high frequency of healthy carriers [1]. This requires uniform population screening and timely diagnosis throughout the country and the need of updated best practice.

## 2. Materials and Methods

The Italian Society of Thalassemia and Hemoglobinopathies (SITE) has undertaken a project aimed at integrating available evidence with expert opinions through a systematic method in order to reach an adequate degree of consensus on recommendations for clinical practice.

These best practices are based on GRADE-ADOLOPMENT methodology [2].

For the literature search, various search strings were built using the following keywords (both as MeSH and non-MeSH terms): thalassemia, sickle, hemoglobinopathies, diagnosis, screening, carrier screening, heterozygosis, heterozygotes, heterozygous, HbA2, MCV, false positive, screening limit, limitation, cut off.

Each search string was filtered for review/guidelines/systematic reviews, and the relevant works in English from the last 15 years were selected. The search was conducted on Pubmed, EMBASE and Cochrane, and the relevant documents available on the WHO, NHS, CDC, ACOG and up-to-date sites were assessed as well.

The search was completed by manually adding “missing papers”.

The team of authors evaluated the full texts of the selected papers and autonomously extracted recommendations based on screening deemed relevant to the writing of this document.

After completing the assessment of the literature review, the team of authors formulated the recommendations.

These recommendations are meant as complementary to the existing guidelines:

- EMQN [3], Significant hemoglobinopathies: guidelines for screening and diagnosis [4];
- The ENERCA recommendations for preconception or antenatal screening, prenatal diagnosis and genetic counseling of hemoglobinopathies [5];
- The joint SOGC-CCMG Opinion for Reproductive Genetic Carrier Screening: An Update for All Canadian Providers of Maternity and Reproductive Healthcare in the Era of Direct-to-Consumer Testing [6];
- UK NHS sickle cell and thalassemia screening program, UK NHS sickle cell and thalassemia handbook for laboratories [7].

The final version of the document was independently revised by 3 experts in the field.

These best practices shall be updated every three years, starting from the date of publication.

## 3. First Level Tests

First level tests consist of hematochemical examinations which are fundamental to identify healthy carriers or those affected by hemoglobinopathy. Once identified, they should be referred to a specialist with documented experience in the field of hemoglobinopathies (internist, hematologist, pediatrician, clinical geneticist) to complete diagnostic investigations and be provided with possible preventative options and adequate support [8].

Hemoglobinopathy carriers should be encouraged to inform family members of the appropriateness to undergo screening in turn [9].

It is important to identify women affected by hemoglobinopathy (drepanocytosis, Hb SC, microdrepanocytosis, nontransfusion-dependent β-thalassemia (NTDT), Hemoglobin H disease) before pregnancy or in the first weeks of pregnancy to adequately monitor the health condition [4].

Neonatal screening may be capable of identifying infants suffering from drepanocytic syndromes (SCD) at an early stage. It can be performed using HPLC or capillary electrophoresis on a blood sample from metabolic screening filter papers or from cord blood. Neonatal screening could identify other hemoglobin variants (e.g., HbE) and HbH disease, thus ensuring adequate family counseling. Some conditions are not detectable by neonatal screening such as being a healthy carrier of alpha and beta thalassemia, whereas others can be suspected because of the absence of HbA [4,10]. Hemoglobin A is generally detectable after a gestational age of 30 weeks; in the case of extreme prematureness, HbA or β-globin variants might not be detectable. In these cases, it is necessary to evaluate the family history. Univocal EBM recommendations regarding the efficacy of neonatal screening are not possible because there are no clinical studies clearly demonstrating a benefit derived from neonatal screening. Data from reviews and economic analyses suggest that neonatal screening allows an early start of clinical check-ups and antibiotic prophylaxis, which has proved to be crucial in reducing mortality from infections in SCD [11]. Therefore, neonatal screening can be locally considered, evaluating the cost/benefit ratio.

The result of screening for hemoglobinopathies does not change over time; therefore, it can be saved and does not have to be repeated in the course of a life.

Although first level diagnostics is, by now, widespread in many general laboratories, the management of complex analytical data (such as the clinically relevant hemoglobin variants homozygous thalassemia or compound heterozygous thalassemia) and the treatment of patients with hemoglobinopathies, in general, remains the prerogative of a few specialized referral clinical centers.

Screening for hemoglobin defects should be required in subjects with: microcytosis with normal iron indices, a family history of hemoglobinopathies, confirmation of a clinical suspicion in the presence of severe anemia, high levels of hematocrit, nonspherocytic hemolytic anemia and in the preconception period or pregnancy. Screening can also be extended to targeted prevention programs, such as at birth in at-risk populations and in blood or bone marrow donors [12].

Some situations do not allow one to properly proceed with first level tests (Table 1).

To appropriately screen the patient, a complete blood count (CBC), iron balance and complete Hb separation (HbA2-HbF-Hb variant) are required. In select cases, the measurement of P50 may be used.

The patient has to be informed of the possibility that the tests may produce an inconclusive report; in these cases, further investigations are warranted.

To obtain the Hb pattern, a blood sample with EDTA anticoagulant is recommended, although most separation methods can also perform the test on Li–heparin and Na–citrate.

Fasting is not essential for a hemoglobin pattern, but it should be considered that first level tests for hemoglobinopathies also include those for the assessment of iron balance for which a lipemic serum is contraindicated.

To determine P50, in addition to arterial blood, venous blood can be used to define a possible increase or decrease in oxygen affinity [13].

It is advisable to perform CBC and evaluation of iron indices within 12/24 h of blood sampling. The examination of the Hb pattern can be performed within seven days after blood sampling by storing the sample at 4 °C, bearing in mind that some unstable hemoglobin variants might not be identifiable in long-stored samples.

The diagnosis of hemoglobinopathies must be entrusted to dedicated personnel experts in hemoglobinopathies [14].

### 3.1. Complete Blood Count (CBC)

The automated measurement of the total number of erythrocytes (RBC), globular indices (MCV: mean corpuscular volume, MCH: mean corpuscular hemoglobin content, MCHC: mean corpuscular hemoglobin concentration), hemoglobin (Hb) and globular distribution (RDW: red cell distribution width, standard deviation of erythrocyte diameter measurement, expressed as a percentage of the mean or as a coefficient of variation) is recommended.

A high number of RBC (about 6–7 × 10^6^/mL) are often present in healthy carriers as a compensation for the chronic reduction in MCH, with no increase in hematocrit. Erythrocytosis is less marked in some conditions such as folic acid deficiency, a vitamin essential in cell divisions. An increase in the number of RBC can be observed in patients with iron deficiency who respond to iron therapy. The RBC count may not be reliable during pregnancy because of hemodilution.

In carriers of hemoglobinopathies, the erythrocyte volume and hemoglobin content are reduced (MCV < 80 fl and MCH < 27 pg in adults; for pediatric patients see below). Pathologic conditions such as a lack of vitamin B12 may cause a false high value of MCV. A false elevation of MCV can also be observed in samples taken more than 24 h before analysis.

**Figure 1:**
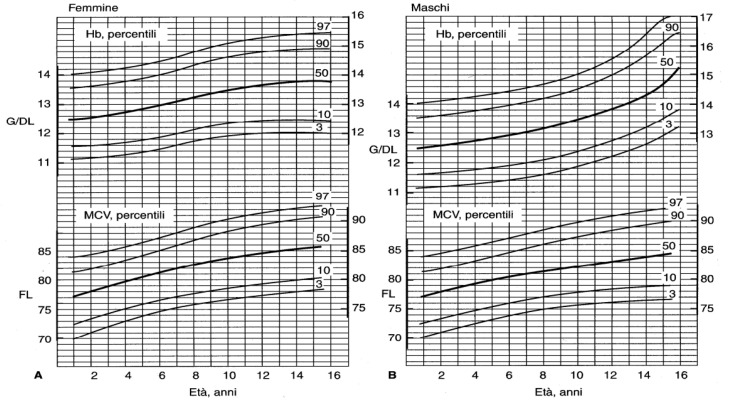
Percentile curves of hemoglobin (Hb) values and mean corpuscular value (MCV) in females (**A**) and males (**B**) in the first 15 years of life (from [16]).

For pediatric ages (up to 16 years), age-related reference values are required to diagnose anemia (<3° centile or −2SD) and microcythemia (<3° centile or −2SD) (Table 2 for the first year of life and Figure 1 up to 16 years).

RDW is a faster and simpler but less complete measure of the degree of anisocytosis than the blood smear. Generally, a wide RDW is observed in iron deficiency, whereas in thalassemia, it is generally normal or only slightly deviates from the norm [17]. RDW may be altered in various heart and liver pathologies [18].

### 3.2. Separation of Hemoglobin

The most widely used method for hemoglobin separation is high performance liquid chromatography (HPLC). HPLC must be a cation exchange apparatus, with a double pump continuous linear gradient and with CV < 5%. As far as the measurement of HbA2 and Hb F is concerned, the instruments available on the market usually have a good performance and provide comparable results in terms of pathology, whereas a greater dispersion of values is observed for HbA2 borderline levels (about 3.3%), that can be diriment for a silent beta trait. Therefore, the use of calibrators is recommended to obtain a high level of accuracy. The capacity to separate and distinguish hemoglobin variants among various systems depends mainly on the type of gradient applied.

Capillary electrophoresis (CE) can be reliable for the screening and confirmation of hemoglobin variants (Table 3). The use of a combination of different techniques can allow a better identification of hemoglobin variants and reduces the probability of artifacts.

Methods for the evaluation of Hb A1c are unreliable for the diagnosis of hemoglobinopathies, and only determination using HCPL or CE can be employed for the first level diagnosis of hemoglobinopathies.

#### Hemoglobin A2

Hemoglobin A2 is detected in normal individuals after six-eight months of age. HbA2 normal values are generally considered between 2.5 and 3.2%. It is worth remembering that values between 2.2 and 2.5 and between 3.2 and 3.5 can be detected in a low percentage of “normal” subjects; in carriers of “mild beta thalassemia” mutations, values between 3.2 and 3.5 can be observed. However, in no cases can diagnosis be based just on hemoglobin A2 without taking erythrocyte indices into account.

HbA2 may be overestimated without the presence of a thalassemic defect (Table 4), whereas it might be up to standard for some rare “silent” thalassemic defects.

Overestimate of HbA2 is a frequent event in the presence of “Hb S type” variants because of the interference with the glycated fraction of the variant itself. In these cases, if the tested subject was not transfused in the past three months and exhibits a relative percentage of the variant lower than 48%, it can certainly be concluded that the variant is present in the heterozygous state. The percentage value obtained must be specified in the report, but the overestimation should be pointed out, after excluding that it is a transfused patient.

Carriers of one α-globin gene mutation (αα/-α) generally display normal levels of HbA2; inactivating the mutation/deletion of two α-globin genes (αα/- -) is related to HbA2 at the lower or slightly reduced limits of the normal range. Concurrent heterozygosity for β-globin mutation can mask the presence of a α-globin gene mutation in first level tests [17].

HbA2 may not be quantifiable in HPLC and in CE due to interference with a Hb variant (Table 5 and Table 6).

### 3.3. Fetal Hemoglobin

Fetal hemoglobin (HbF) is the main hemoglobin fraction during fetal life and accounts for approximately 80% of total hemoglobin at birth. After birth, HbF synthesis gradually decreases and is replaced by HbA; from the second year of life, HbF levels are usually below 1%.

HbF quantification is useful in several globin gene disorders, e.g., transfusion-dependent beta thalassemia, heterozygous δβ-thalassemia. Elevated HbF in adults can be frequently caused by the hereditary persistence of fetal hemoglobin (HPFH), a benign condition without clinical and laboratory signs. Some acquired conditions can induce a slight increase in HbF (Table 7).

A slight HbF increase (1–3%) can be observed during pregnancy; in suspect cases, it is recommended to repeat the assay 6 months after delivery.

(δβ)0 thalassemia usually display normal/low HbA2 levels and increased HbF levels (5–20%). These forms can be distinguished from HPFH based on normal hematological parameters in HPFH [19].

Measurement of HbF is also required to monitor sickle cell anemia treatment with drugs that can induce its production.

HbF quantified by CE may be partially underestimated for values lower than 1.5%, whereas in HPLC, it may be overestimated if partially superimposed to the labile fraction of glycated Hb or, with some systems, may not be correctly quantified for high values.

Interference in the assessment of HbF are few: with HPLC, a superimposition with the variants Hb Marseille and Hb J Iran can be observed, whereas in CE, there is a superimposition with Hb Richmond, Hb G San Josè, Hb Presbyterian and Hb Porto Alegre.

The evaluation of HbA2 and HbF in the first year of life, especially in the first 6 months, requires the consultation of age-appropriate reference tables (Table 8 and Table 9).

### 3.4. Hb Variants

The hemoglobin variants described so far are mainly caused by mutations in beta and alpha globin chains and in most cases, differ from HbA structurally because of the replacement of one amino acid as the consequence of the mutation of a single nucleotide base.

The great majority of hemoglobin variants are not associated with abnormal hematological parameters, e.g., hemoglobin S (Hb S). As a consequence, hemoglobin fraction assay is recommended for proper carrier diagnosis, in particular, if the ethnic origin is from areas with a high frequency of these variants.

Several rare variants have relevant clinical features, such as altered oxygen affinity (especially high affinity). For a first level laboratory, these cases may be challenging to recognize because they require knowledge of specific anamnestic information and the use of molecular analysis for diagnosis with certainty.

The separation techniques normally used in a first level laboratory allow the identification of about two/three of the known variants, recognizing among them the relevant and most common abnormal hemoglobin (Hb S, C, E, Lepore) with an acceptable—but still presumptive—degree of specificity. In any case, characterization through molecular analysis is recommended, especially for those variants with a less specific chromatographic or electrophoretic pattern. The relative percentage and elution time of the variant in HPLC or CE allows one to hypothesize the change in the electric charge which occurred and the type of globin chain involved. The relative percentage of most structural hemoglobinopathies, in a heterozygous state, varies between 15 and 45% when the defect affects beta chains and between 5 and 30% when it affects one alpha globin chains. These percentages depend on the stability of the mutated chain, affinity with the normal homologous chain to form a stable tetramer, possible reduced synthesis of the globin variant, the number of mutated structural genes out of the normal number of genes present (two for beta chains and four for alpha chains), the oxygen affinity of mutated hemoglobin or association with alpha or beta thalassemia defects.

When a variant, whatever it is, has a relative quantity greater than 50%, the presence of a double defect of the same gene on two different alleles is certain if the subject examined has not been recently transfused. In these cases, the molecular analysis (second level) is mandatory to confirm the correct diagnosis. The presence of an alpha thalassemia mutation associated with a beta globin variant reduces the percentage of the variant compared to what is expected (e.g., HbS approx. 35–40% with αα/α- genotype and HbS < 35% with αα/-- or α-/α- genotype).

HPLC and CE provide some indications for the identification of variants. These messages only suggest a diagnostic hypothesis that can never be considered certain and consequently transferred into the report without validation by at least one confirmatory test. The most frequently performed confirmatory test is molecular analysis; in some cases, HbS is confirmed using a sickling test.

In patients with suspected vaso-occlusive crisis, rapid screening for drepanocytosis in emergency contexts can be used. This test should only be utilized in emergency situations and must be followed by first and second level confirmation tests.

### 3.5. Iron Parameters and Hemoglobinopathies

Iron deficiency is the major cause of anemia and, as is known, reduces MCV, MCH and total hemoglobin. Contrary to a thalassemia healthy carrier, in iron deficiency, the red blood cell (RBC) count is often normal or reduced. In order to appropriately distinguish iron deficiency and thalassemia carriers, iron parameters (serum iron, ferritin, transferrin and the saturation index—SI) should be screened.

In the case of iron deficiency, the screening of hemoglobinopathies should only be performed after correcting the iron balance and, unless urgent, after an adequate period of time to allow restoration of the erythrocyte population.

The quantification of reticulocyte Hb can be a reliable marker of hemoglobin content and can be used to identify iron deficiency [22].

#### Recommendations

- Adequate counseling with a specialist with documented experience in the treatment of hemoglobinopathies (internist, hematologist, pediatrician, clinical geneticist) should be offered to all hemoglobinopathy carriers in order to explain the probabilities of disease transmission, the pathology, the state-of-the-art treatment and the possibilities of prenatal diagnosis [6].
- First level tests for hemoglobinopathies (CBC, iron parameters and HPLC and/or CE) should be delayed if the patient has received a red blood cell transfusion in the previous three months or in the case of iron deficiency.
- First level tests for diagnostic certainty of alpha+ thalassemia are not sufficient. (Diagnostic certainty can only be obtained by molecular analysis.)
- At birth, first level examinations for the diagnosis of heterozygous beta thalassemia, Hb Lepore, or delta beta thalassemia are not sufficient for diagnostic certainty.
- CBC and iron indices should be analyzed within 12/24 h of blood sampling.
- Hb pattern analysis can be carried out within seven days of blood sampling test in EDTA by storing the sample at 4 °C. In the case of a suspected unstable variant, the examination should be performed as soon as possible to avoid degradation of the variant.
- Values of MCV < 80 fl and of MCH < 27 pg should generally be considered diagnostic of microcythemia in adults; age-related normal values should be considered in subjects <16 years (Table 2 and Figure 1).
- HPLC and/or CE are recommended for the screening of hemoglobinopathies. Both methods can be mutually used as confirmatory tests. To separate Hb fractions using HPLC, it is essential to use a cation exchange apparatus, with a double pump continuous linear gradient and with a variation coefficient of <5%.
- When interpreting HbA2, consider the presence of factors that might over- or underestimate it (see Table 3).
- The presence of HbF above normal values beyond the first year of life should be investigated with second level tests if associated with moderate microcytic anemia and/or splenomegaly.

## 4. Second Level Test

### 4.1. Molecular Analysis

Molecular analysis is used in:

- subjects with nondiriment first level tests to confirm or refute the diagnosis of a healthy carrier. To correctly identify α+-thalassemia carriers, molecular analysis is required [23]. Carriers of very mild or silent mutations of the β-globin gene, in which chain production is only minimally reduced, may have normal hematological parameters and are only recognizable by molecular analysis [17];
- suspected carriers of both α and β thalassemia to correctly define the thalassemia recurrence risk;
- the definition of the mutation responsible for the carrier status on the indication of a specialist in the field;
- sideropenic pregnant couples. It is preferable to search for alpha- and beta globin gene mutations if one partner is already known to be a beta thalassemia carrier before the normalization of iron levels in order to avoid the delay of a prenatal diagnosis if necessary [23].

It is preferable to use DNA extracted from peripheral blood taken in EDTA.

Other sources which may be used in special cases:

  ○ DNA can be extracted by a saliva sample from children for whom peripheral blood sampling is difficult;
  ○ buccal swabs from bone marrow transplant recipients. (Note: a saliva sample might not allow a definitive report due to the presence of the leucocytes of the donor in the saliva).

For molecular diagnosis, several DNA analysis techniques are used (Table 10). Not all techniques are able to detect all mutations of globin genes (Table 11), and the choice of the technique to be used must be based on the type of mutation in order to minimize the risk of a false negative result.

Due to their biological structure, α-globin genes are prone to deletions/duplications, whereas the β-globin gene is more susceptible to point mutations [24].

NGS can be considered as an alternative to Sanger sequencing, following the recommendations for NGS diagnostics.

If borderline HbA2 levels are detected, it is recommended to rule out β+ mutations and α globin gene triplication/quadruplication.

Genotype–phenotype correspondence should always be verified.

During counseling, it should be clearly specified that first level tests can only exclude the risk of transfusion-dependent thalassemia or sickle cell syndrome, conditions for which prenatal diagnosis (PND) is indicated (Table 12). A more complete assessment of the risk of NTDT recurrence, conditions for which PND is not indicated (Table 12), can be achieved through a second level test, ruling out β+ mutations and α globin gene triplication/quadruplication in the partners of beta globin gene mutation carriers.

#### Recommendations

- DNA analysis should always be performed in couples at risk (both partner carriers of hemoglobinopathies and/or triplication-quadruplication of α globin genes) in cases where the first level tests are not decisive or in couples where one partner is a carrier of alpha- and the other of beta thalassemia in order to exclude the possibility that the beta trait is masking an alpha trait, thus underestimating the risk of HbH in the fetus or vice versa.
- In the case of sideropenia in pregnant couples, it is preferable to start molecular analysis before iron level normalization to avoid a delay in a prenatal diagnosis if necessary [23].
- In the case of suspected α thalassemia, techniques such as reverse dot blot or GAP-PCR can be used as first level screening for recurrent α globin gene mutations. In negative cases with strong clinical suspicion of α thalassemia carrier, it is recommended to proceed with second level tests such as direct sequencing or MLPA.
- Direct sequencing of the HBB gene is recommended.
- In laboratories equipped with NGS, this can be considered as an alternative to Sanger sequencing, following the recommendations in force for NGS genetic diagnosis.
- For subjects with HbA2 borderline levels, it is recommended to rule out β+ mutations and α globin gene triplication/quadruplication.
- Genotype–phenotype correspondence should always be verified.
- During counseling, it should be clearly specified that first level tests can only exclude the risk of transfusion-dependent thalassemia or sickle cell syndrome, conditions for which prenatal diagnosis is indicated (Table 12).

### 4.2. Prenatal Diagnosis (PND)

All couples at risk of hemoglobinopathies should be offered a consultation with specialists in the field in order to discuss the different reproductive options and the possibilities to access a prenatal diagnosis.

As with all genetic counseling, discussions should be nondirective, avoid jargon and incorporate the cultural beliefs of the couple where possible. The final decision must be made by the couple without external influences [25].

Counseling should be offered prenatally and/or in the first weeks of pregnancy to allow time for the couple to make an informed choice and not to preclude the possibility of access to some prenatal diagnosis techniques. (Ideally, prenatal diagnostics should be completed within 12–14 weeks) [28].

To allow early identification of couples at risk, screening of both partners simultaneously instead of performing a sequential screening is useful. The request for screening tests should be made at the first obstetric visit if the couple has not undergone previous investigations.

Early diagnosis of the possible presence of alpha thalassemia with the risk of fetal hydrops is fundamental to ensure the correct pregnancy monitoring (intrauterine transfusion, risk to mothers of generalized edema, Ballantyne syndrome or mirror syndrome [29], possible future stem cell transplant).

Five experts from five Italian centers were consulted for prenatal diagnosis recommendations. The expected phenotype was proposed based on data in the literature, and the experts consulted and agreed on the proposed definition [3,10]. For each indication, the degree of agreement among the experts is indicated.

When prenatal diagnosis is not performed, testing the infant within 3–6 months of life is recommended in order to ensure early access to appropriate treatments if needed.

PND should follow dedicated procedures with coordination among the maternity ward, laboratory, and microcythemia center to optimize the sample and report management.

Exclusion of maternal–fetal contamination is mandatory [25].

Fetal karyotype analysis should be performed if sufficient material is available [3,25].

The PND report should be delivered as quickly as possible in dedicated genetic counseling [26].

If the father-to-be refuses to undergo carrier testing, it should be specified that fetal DNA analysis could indirectly determine his genotype for globin genes [27].

If the father-to-be is not available, it is possible to perform PND to evaluate the risk of major hemoglobinopathies in the fetus, after specialist advice is given to the woman who should be made aware of the benefits and risks of this approach. If fetal DNA analysis shows the presence of the maternal β-globin gene mutation, the sequencing of the whole gene is mandatory in order to rule out the presence of a second mutation [25]. The report should specify that the paternal genotype is unknown and that fetal genetic analysis is not able to exclude the presence of all hemoglobinopathies, preferably indicating the residual risk of hemoglobinopathies in the fetus.

For twin pregnancies, it is fundamental to obtain an accurate diagnosis in each twin (e.g., through STR analysis).

The molecular analysis techniques are the same as those in use for postnatal diagnosis; the laboratory will adopt the most suitable technique, among those locally available, to search for parental mutation.

It is considered useful to confirm the PND result after birth using cord blood or by dedicated sampling in the first year of life in the case of an unaffected fetus; in the case of an affected fetus, it is recommended to test the patient within the first 3 months of life [25].

Chorionic villus sampling (CVS) is the procedure of choice for PND because it allows one to obtain good quality fetal DNA between 10 and 12 weeks of gestation [30].

Amniocentesis is proposed when CVS cannot be performed due to technical issues (e.g., posterior placenta) in advanced pregnancies (Amniocentesis is performed after 16 weeks of gestation).

Cordocentesis: Funicular blood sampling can be used both for biochemical analyses (CBC–hemoglobin separation) and molecular tests. It can be performed at a late stage of pregnancy (after 18–20 weeks of gestation) and has a higher risk of miscarriage (about 2%) than other PND techniques; for this reason, it tends to be used less frequently than in the past, even in the diagnosis of fetal hydrops, since diagnosis is more frequently made using a molecular test in the first trimester or by ultrasound diagnosis, that detects cardiomegaly and placentomegaly starting from the second trimester. Experienced ultrasonographers can already recognize this condition at a gestational age of 11–13 weeks [25].

Celocentesis: Molecular testing after celomic fluid aspiration can be performed from the eighth week of gestation; the test is performed on DNA extracted from fetal erythroblasts in the celomic fluid [31]. This technique is not widespread and is performed only by one center in the world.

Couples at risk of fetal hydrops who refuse PND/where PND is not possible should be referred for fetal ultrasound evaluation to assess the cardiothoracic index, placental thickness or middle cerebral artery peak systolic velocity [6,26,27,29,31]. The cardiothoracic ratio seems to be the most reliable method for early recognition of fetal hydrops. Diagnosis of an affected fetus is suggested by: a cardiothoracic ratio >0.5 before 17 weeks of gestation; a placental thickness >18 mm before 15 weeks of gestation or >30 mm at ≥18 weeks of gestation or higher than average by more than 2 SD for the gestational age; or by a middle cerebral artery peak systolic velocity >1.5 multiples of the median (MoM) for the gestational age after 15 weeks [32].

### 4.3. Cell-Free Fetal DNA (cffDNA)

There is no evidence that this technique is so sensitive and specific to warrant its exclusive use in the prenatal diagnosis of recessive diseases [33,34]. Studies are being conducted for the diagnosis of recessive diseases on cffDNA through the haplotype reconstruction of mutated allele, but these techniques have not yet been validated for diagnostics. At present, cffDNA analysis in the case of carrier parents can be aimed only at confirming/excluding the presence of a paternal mutation.

### 4.4. Preimplantation Genetic Testing (PGT)

Preimplantation genetic testing is a very specialized topic; therefore, couples deciding to undergo PGT are advised, after genetic counseling with an expert in hemoglobinopathies, to contact an experienced assisted reproduction center where the operating methods of PGT, with its benefits and risks, can be explained. If the couple opts for PGT after getting pregnant, PND or a subsequent follow-up of the infant is recommended [26].

### 4.5. Gamete Donation

It is recommended to screen the individual/couple requesting gamete donation for hemoglobinopathies. In the case of a suspected carrier of hemoglobinopathies, it is necessary to perform carrier tests in the gamete donor.

As a general rule, it would be preferable to know the possible status of the carrier in the gamete donor.

#### 4.5.1. Recommendations

- At-risk couples should receive counseling with a specialist (internist, hematologist, pediatrician, clinical geneticist) in the treatment of hemoglobinopathies.
- Screening for hemoglobinopathies should be routinely offered as part of the examinations offered during preconceptual screening and before medically assisted procreation [4]. In the case of gamete donation, the donor should undergo screening for hemoglobinopathies [4].
- Couples for whom prenatal counseling has not been possible should be informed of the risk of recurrence and of PND possibilities for future pregnancies [6].
- It is preferable to perform PND through CVS in the first trimester of gestation.
- Before PND, the genotype of both parents must be determined.
- If the phenotype of the father is not available, PND limits should be clearly explained (e.g., possible failure to detect a second mutation of the β-globin gene because of technical limitations).
- Even if PND has been performed, it is preferable to perform a carrier test in the child, ideally in the first year of life, in the case of an unaffected fetus; in the case of an affected fetus, it is recommended to examine the patient within 3 months of life.
- In all at-risk couples in whom a prenatal diagnosis is not made, it is mandatory to perform a carrier test in the infant within 3–6 months of life in order to ensure appropriate treatment.
- In the case of gamete donation/preimplantation genetic testing, specific advice from an experienced center is recommended.
- With the techniques currently available in diagnostics, the analysis of cell-free fetal DNA (cffDNA) is not sufficient to diagnose fetal hemoglobinopathies.

#### 4.5.2. Informed Consent to Investigations

In all cases of DNA analysis (pre- and postnatal), written consent must be obtained from the patient or his/her guardian as per current regulations [15].

#### 4.5.3. Medical Reporting

Medical reporting should only be undertaken when the results of all tests are available. The report should be written in a clear manner, with a short comment on the result and a suggestion to refer carriers or the affected persons for specialist advice. The report should include technical data and, at the end, a clear comment with any recommendations.

The report should explicitly specify whether blood transfusions have been performed in the previous 4 months since a recent transfusion might cause a false interpretation of laboratory data [4].

A final report should be prepared by the consulting specialist in which the main results of the examinations and the conclusions on carrier or affected status are specified, with the relevant recommendations for couple screening or for prenatal diagnosis and clinical follow-up when indicated. The report should use terminology that is easily understandable by the patient.

## Figures and Tables

**Table 1:** Conditions where 1st level test would be inappropriate.

Conditions Where 1st Level Test Would Be Inappropriate
(a) In the three months following a transfusion, if not intended for monitoring in polytransfused patients
(b) In recently ascertained iron deficiency (*)
(c) In therapy for severe unresolved iron deficiency (*)
(d) In subjects in whom beta Thalassemia has already been ruled out and you want to exclude alpha Thalassemia too
(e) At birth for heterozygous beta Thalassemia, Hb Lepore and delta-beta Thalassemia

(*) Except for those cases where the clinical context urgently requires to exclude an alpha (with normal or decreased HbA2) or beta (increased HbA2) thalassemia mutation, bearing in mind that the data obtained has no conclusive value and should be evaluated critically at the end of therapy, repeating the test especially if HbA2 was normal or reduced.

**Table 2:** Normal values of hemoglobin (Hgb g/dL), hematocrit (Hct%), Red blood cell count (RBC 10^12^/L), mean corpuscular hemoglobin (MHC pg), mean corpuscular volume (MCV fl) and mean corpuscular hemoglobin concentration (MCHC g/dL) *.

*n*	Age (Months)						
	0.5 (*N* = 232)	1 (*N* = 240)	2 (*N* = 241)	4 (*N* = 52)	6 (*N* = 52)	9 (*N* = 56)	12 (*N* = 56)
Hgb (mean ± SE)	16.6 ± 0.11	13.9 ± 0.10	11.2 ± 0.06	12.2 ± 0.14	12.6 ± 0.10	12.7 ± 0.09	12.7 ± 0.09
−2 SD	13.4	10.7	9.4	10.3	11.1	11.4	11.3
Hct (mean ± SE)	53 ± 0.4	44 ± 0.3	35 ± 0.2	38 ± 0.4	36 ± 0.3	36 ± 0.3	37 ± 0.3
−2 SD	41	33	28	32	31	32	33
RBC count (mean ± SE)	4.9 ± 0.03	4.3 ± 0.03	3.7 ± 0.02	4.3 ± 0.06	4.7 ± 0.05	4.7 ± 0.04	4.7 ± 0.04
−2 SD–+2 SD	3.9–5.9	3.3–5.3	3.1–4.3	3.5–5.1	3.0–5.5	4.0–5.3	4.1–5.3
MCH (mean ± SE)	33.6 ± 0.1	32.5 ± 0.1	30.4 ± 0.1	28.6 ± 0.2	26.8 ± 0.2	27.3 ± 0.2	26.8 ± 0.2
−2 SD	30	29	27	25	24	25	24
MCV (mean ± SE)	105.3 ± 0.6	103.1 ± 0.3	94.8 ± 0.3	86.7 ± 0.8	76.3 ± 0.6	77.7 ± 0.5	77.7 ± 0.5
−2 SD	88	91	84	76	68	70	71
MCHC (mean ± SE)	314 ± 1.1	318 ± 1.2	318 ± 1.1	327 ± 2.7	350 ± 1.7	349 ± 1.6	343 ± 1.5
−2 SD	281	281	283	288	327	324	321

* These values were obtained from a group of 256 healthy full-term infants followed at the Helsinki University Central Hospital. Infants were receiving iron supplementation and had normal Ievels of transferrin saturation and serum ferritin. Values at the ages of 0.5, 1 and 2 months were obtained from the whole cohort; at higher ages, subjects with iron deficiency were excluded. From [15].

**Table 3:** Common variants diagnosis.

Test	Specific Test	Confirmation Test	Other Test	Comments
HPLC	X	X		Separation and quantification
CE	X	X		Separation and quantification
Complete blood count	X			EvalueteS RBC indices: G.R (X103/pL), Hb (g/dL), mCV (fL), MCH (pg), Ht (%).
Serum iron	X			In alternatives ZnPP.
Transferrin	X			
Ferritin	X			
Reticulocytes			X	(%)
P50				Arterial or venous blood test in the presence of an increased hematocrit (<45% in women and> 50% in men)
Haptoglobin			X	If haemolytic anemia is present
Methemoglobinemia			X	If cyanosis is present
Bilirubin			X	If haemolytic anemia is present

**Table 4:** Main individual preanalytical variables that can modify the value of HbA2.

Increased HbA2	Reduced HbA2
Hyperthyroidism	Severe Iron-deficiency anemia
Megaloblastic anaemia	Sideroblastic anemia
Antiretroviral therapy for HIV	δ-Thalassaemia (b)
Hb unstable variants	δ -Thalassaemia chain variants (c)
Supernumerary alpha genes variants (i.e., ααα/αα)	α-Thalassaemia chain variants(d)
Glycated component of the Hb B variant if present (a)	Same type of HpFH due to defect in the Gamma gene promoter (e)
Liver disease/alcohol	δβ-Thalassaemia
Hypertrophic osteoarthropathy	α-Thalassaemia: borderline in Alf + or ALPHA while it is marked in H hemoglobinosis
KLF1 mutations	Hb Lepore (f)
	HbD (a) e C (f)

(a) In the case of quantification with HPLC systems. (b) The value of HbA2 could be normal. (c) The real value of HbA2 is equivalent to about twice the value measured and defined as such by the instrumentation. In the event that the HbA2x is detected, the real HbA2 is given by the sum of the two fractions. (d) In this circumstance, the value of HbA2 must be added to the value of HbA2x. (e) This is what occurs in the so-called “Sardinian delta-beta thalassemia” with the mutation −196 C>T of the promnoter of the Ay gene. (f) Detectable only in the case of quantification with CE.

**Table 5:** Examples of hemoglobin variants that co-elute with or near HbA2 in HPLC.

Hb Abruzzo	Hb Kenya
Hb Akron	Hb Korle Bu *
Hb Boras	Hb Lepore Baltimore
Hb Bethesda *	Hb Lepore Boston
Hb Chandigarth	Hb Lepore Hollandia
Hb Deer Lodge	Hb Loves Park *
Hb D Iran *	Hb M Saskatoon
Hb Denver *	Hb Muravera
Hb D-Ouled Rabah	Hb Nebraska
Hb E	Hb Ocho Rios
Hb Ethiopia *	Hb Osu Christiansborg *
Hb Fort Worth	Hb Paddington
Hb G Copenhagen	Hb Rocky Mountain
Hb G Coushatta *	Hb San Bruno *
Hb G Ferrara	Hb Santa Juana *
Hb G Galveston	Hb SId (the aged adduct of Hb due to glutathione)
Hb G Honolulu *	Hb Spanish Town
Hb G Taipei	Hb Toulon
Hb Hoshida	Hb Tubingen
Hb Hamadan	Hb Zuri
HPLC, high-performance liquid chromatography.	

* These haemoglobin variants are sometimes labelled HbA2 and sometimes unknown depending on the instrument and slight variations in retention times. Even when the variant does not precisely co-elute with HbA2, it may still interfere with the HbA2 measurement and so a different method of measuring the HbA2 should be used in this situation.

**Table 6:** Examples of hemoglobin variants that migrate with or near HbA2 in capillary electrophoresis.

Hb Chad
Hb E-Saskatoon
Hb O-Arab
Hb C Harlem *

* Insufficient separation for accurate quantification.

**Table 7:** Conditions in which an increase in HbF not ascribable to gamma globin defects can be detected.

Congenital or acquired anemias from primitive bone marrow failure with or without displasia	Neoplasias	Conditions associated with specific therapeutic treatments	Other conditions
Congenital or acquired aplastic anemia	Hepatocarcinoma	Chemotherapies for leukemias	Monoclonal gammopathy of uncertain significance
Megaloblastic anemia from vitamin deficiency	Myeloid acute leukemias	Therapy with hydroxyurea, butyrates and erythropoiesis-stimulating agents	Pregnancy
Diamond-Blackfan anemia	Primitive myelofibrosis		Chronic renal insufficiency
Some forms of normoblastic anemia	Juvenile chronic myelomonocytic leukemia		Hyperthyroidism
Congenital sideroblastic anemias			Trisomy 13
Acquired sideroblastic anemias			
Nocturnal paroxysmal hemoglobinuria			

**Table 8:** Values of HbA2, HbF and some relevant erythrocyte indices in normal infants and in beta thalassemia carriers during the first year of life.

Age	Subject	Number	HbA2 (%)	HbF (%)	Hb (g/dl)	MCV (fl)	MCH (pg)
At birth	Normal β Thalassaemia	16 31	0.4 (0.2) 0.5 (0.2) NS	65.1 (7.5) 73.8 (10.1) *p* < 0.05	18.1 (2.3) 18.3 (2.3) NS	101.3 (6.9) 98.5 (8.1) NS	35.1 (3.5) 33.8 (2.6) NS
3 months	Normal β Thalassaemia	8 12	1.7 (0.3) 3.2 (0.7) *p* < 0.01	18.1 (3.6) 27.0 (10.5) *p* < 0.05	11.0 (0.7) 10.0 (1.1) NS	82.5 (3.6) 69.9 (5.8) *p* < 0.001	27.9 (2.0) 22.8 (1.8) *p* < 0.001
6 months	Normal β Thalassaemia	8 10	2.5 (0.3) 4.8 (0.7) *p* < 0.001	3.2 (1.1) 8.2 (4.0) *p* < 0.001	11.5 (0.8) 10.5 (0.8) *p* < 0.05	74.7 (2.9) 59.2 (3.5) *p* < 0.001	24.9 (1.4) 19.2 (1.2) *p* < 0.001
9–10 months	Normal β Thalassaemia	6 14	2.5 (0.4) 5.1 (0.5) *p* < 0.001	2.6 (1.4) 4.4 (2.1) NS	12.5 (1.0) 11.1 (0.9) *p* < 0.005	76.8 (5.2) 58.7 (1.6) *p* < 0.001	25.9 (1.7) 19.6 (0.9) *p* < 0.001
1 year	Normal β Thalassaemia	5 8	2.5 (0.3) 4.8 (0.4) *p* < 0.001	1.4 (0.6) 4.1 (2.1) *p* < 0.02	12.3 (1.0) 11.2 (0.9) *p* < 0.005	74.6 (5.0) 57.5 (2.4) *p* < 0.001	24.8 (2.7) 18.7 (0.9) *p* < 0.001

Table adapted from [20]. Values are means (SD); the statistical significance between subjects was tested by one-tailed Student t test. Hb, haemoblobin; MCH mean cell haemoglobin, mean cell volume; NS not significant.

**Table 9:** HbA, HbF (±SD), red blood cells (RBC) before and after separation in density gradient of neocytes (N) and gerocytes (G) during the 1st year of life.

Age	Number of Subjects	HbA			HbF		
		N	G	RBC	N	G	RBC
At birth	30	0.64 ± 0.15	0.42 ± 0.07	0.49 ± 0.12	54.0 ± 8.8	72.0 ± 9.2	66.0 ± 7.6
1 month	10	0.91 ± 0.25	0.40 ± 0.12	0.72 ± 0.29	44.0 ± 5.7	68.0 ± 6.2	52.0 ± 5.5
2 months	6	2.07 ± 0.29	0.96 ± 0.15	1.14 ± 0.32	24.3 ± 6.8	37.5 ± 6.2	33.0 ± 6.5
3 months	8	2.13 ± 0.32	1.35 ± 0.19	1.55 ± 0.31	18.5 ± 5.5	25.6 ± 4.8	21.0 ± 6.1
4 months	8	2.27 ± 0.26	1.72 ± 0.25	1.93 ± 0.28	8.0 ± 3.3	13.6 ± 3.6	10.5 ± 3.5
5 months	6	2.39 ± 0.26	1.86 ± 0.30	2.18 ± 0.21	3.2 ± 1.1	5.6 ± 0.9	4.6 ± 0.9
6 months	6	2.50 ± 0.22	2.10 ± 0.32	2.25 ± 0.30	2.6 ± 0.4	3.9 ± 1.1	3.3 ± 0.9
7 months	5	2.50 ± 0.28	2.19 ± 0.31	2.28 ± 0.27	1.7 ± 0.5	3.3 ± 1.2	2.8 ± 1.1
8 months	5	2.48 ± 0.32	2.25 ± 0.29	2.34 ± 0.36	1.2 ± 0.4	2.3 ± 0.5	1.9 ± 0.7
9–10 months	5	2.53 ± 0.26	2.30 ± 0.28	2.42 ± 0.34	1.1 ± 0.6	2.2 ± 0.6	1.7 ± 0.4
11–12 months	5	2.61 ± 0.24	2.50 ± 0.28	2.53 ± 0.29	1.0 ± 0.3	2.0 ± 0.5	1.4 ± 0.4
2–12 years	20	2.64 ± 0.28	2.56 ± 0.24	2.60 ± 0.30	0.4 ± 0.2	0.7 ± 0.3	0.6 ± 0.2

From [21].

**Table 10:** Advantages and disadvantages of different techniques of DNA analysis [3,24,25].

Method	Advantages	Limits
known mutations detection (Reverse dot blot hybridization, ASO, allele-specific PCR—ARMS PCR, GAP-PCR)	economic easy to perform rapid possible use of commercial kits	can recognize only known mutations may not be standardizable for some specific mutations generally non–“high-throughput” if not using commercial kits, it must be validated risk of allele drop-out
Sanger Sequencing	economic platform utilizable for different analyses can detect mutations in the whole gene	dedicated personnel requires specific expertise not posssible a conclusive diagnosis of large deletions
Pyrosequencing	rapid easy to perform	allows to perform short sequences of DNA (20–50 nucleotides) dedicated personnel specific expertise in the analysis not very widespread equipment
Next generation sequencing (NGS)	utilizable for different analyses can detect mutations in the whole gene sequence allows gene panel analysis	dedicated personnel specific expertise in bioinformatics analyses high costs
High Resolution Melt Analysis (HRMA)	rapid sensitive “high-throughput” can be used also for other analyses	technically more difficult to design the sample in some cases diagnostic confirmation by another method is required dedicated personnel requires specific expertise high costs of the instrument
Real Time PCR	allows to identify qualitative and quantitative variations rapid “high-throughput”	requires specific expertise high costs of the instrument
MLPA	simple, rapid validated commercial kits can detect any copy number variant at the locus	quality and concentration of DNA are critical instrumentation with dedicated personnel specific expertise
arrayCGH	can detect any copy number variant at the locus	specific kits for globin loci does not allow precise characterization of deletion/duplication breakpoints dedicated personnel specific expertise potentially high costs

**Table 11:** Types of mutation identifiable by different DNA analysis techniques [3,26].

Reverse dot blot hybridization, ASO, allele specific PCR—ARMS PCR	Known point mutations/deletions of α and β globin genes
GAP-PCR	Known deletions/duplications of α and β globin genes. Note: amplification of GC-rich regions may be difficult, risk of allele drop out: not recommended in PND
Sanger Sequencing/NGS	point mutations in the whole sequence of α and β globin genes
High Resolution Melt Analysis (HRMA)	point mutations in the whole sequence of α and β globin genes
MLPA	Deletions/duplications in the whole α and β globin gene locus
arrayCGH	Deletions/duplications in the whole α and β globin gene locus

**Table 12:** Prenatal diagnosis orientation table.

Genotype	Expected Phenotype	Degree of Uncertainty in Predicting Phenotype	Agreement among Experts	Indication to Make PND Available	Agreement among Experts
2 severe β0 or β+ mutations	thalassemia major	low	100%	strong	94%
Hb Lepore + severe β0 or β+ mutations	thalassemia major	low	100%	strong	94%
δβ0 + severe β0 or β+ mutations	severe thalassemia intermedia/thalassemia major	low	100%	strong	94%
HbE + severe β0 or β+ mutations	severe thalassemia intermedia/thalassemia major	average	100%	strong	94%
Hb O-Arab + severe β0 or β+ mutations	thalassemia intermedia/thalassemia major	average	94%	strong	94%
Homozygous Hb Lepore	thalassemia intermedia/thalassemia major	low	100%	strong	94%
Homozygous HbS	drepanocytic syndrome	high	96%	clear	90%
Heterozygous HbS + HbC/ Hb O-Arab/ HbD-Punjab	drepanocytic syndrome	average	100%	clear	94%
Homozygous α0 thalassemia	fetal hydrops	low	100%	absolute	100%
2 mild β+ mutations	thalassemia intermedia	average	84%	open	98%
Homozygous δβ0 thalassemia	thalassemia intermedia	low	100%	open	100%
δβ0 + mild β+ mutations	thalassemia intermedia	high	88%	open	98%
δβ0 + Hb Lepore/HbE/Hb O-Arab	thalassemia intermedia	average	88%	open	100%
HbC + severe β0 or β+ mutations	thalassemia intermedia	average	88%	open	100%
Homozygous HbC /HbE/HbD Punjab/Hb O-Arab	thalassemia intermedia	high	88%	low	98%
Hb D-Punjab/Hb O-Arab + severe β0 or β+ mutations	thalassemia intermedia	high	94%	clear	88%
HbS/HbE	drepanocytic syndrome with intermediate course	average	88%	open	94%
HbS + severe β0 or β+ mutation	drepanocytic syndrome	low	88%	clear	100%
HbS + mild β+ mutations	drepanocytic syndrome with intermediate course	average	100%	open	92%
HbS +δβ0 or Hb Lepore	drepanocytic syndrome with intermediate course	average	100%	open	100%
HbS + HbD Punjab	drepanocytic syndrome	high	100%	clear	100%
α0 + α+ thalassemia (--/-α)	HbH disease	average	94%	open	100%
ααα o αααα + severe β0 or β+ mutations	thalassemia intermedia with variable clinical picture	average	86%	open	94%
ααα o αααα + β+ mutation	mild thalassemia intermedia	average	82%	low	100%
2 silent β+ mutations	very mild thalassemia intermedia	low	100%	low	96%
HbC + mild β+ mutations	mild thalassemia intermedia	average	84%	low	92%
HPFH	not clinically significant	low	100%	none	100%
Homozygous α+ thalassemia	not clinically significant	low	100%	none	100%
Homozygous ααα	not clinically significant	low	100%	none	100%

Five experts of five Italian centers were consulted for prenatal diagnosis recommendations. The expected phenotype was proposed based on data in the literature and the experts consulted agreed with the proposed definition [2,27]. For each indication the degree of agreement among the experts is indicated.

## Data Availability

Not applicable.
